# Design and Integration for High Performance Robotic Systems Based on Decomposition and Hybridization Approaches

**DOI:** 10.3390/s17010118

**Published:** 2017-01-09

**Authors:** Dan Zhang, Bin Wei

**Affiliations:** 1 School of Mechanical, Electronic and Control Engineering, Beijing Jiaotong University, Beijing 100044, China; 2 Department of Mechanical Engineering, York University, 4700 Keele Street, Toronto, ON M3J 1P3, Canada; binwei28@yorku.ca

**Keywords:** decomposition and integration, robotic systems, synthesis design, dynamic balancing, control design

## Abstract

Currently, the uses of robotics are limited with respect to performance capabilities. Improving the performance of robotic mechanisms is and still will be the main research topic in the next decade. In this paper, design and integration for improving performance of robotic systems are achieved through three different approaches, i.e., structure synthesis design approach, dynamic balancing approach, and adaptive control approach. The purpose of robotic mechanism structure synthesis design is to propose certain mechanism that has better kinematic and dynamic performance as compared to the old ones. For the dynamic balancing design approach, it is normally accomplished based on employing counterweights or counter-rotations. The potential issue is that more weight and inertia will be included in the system. Here, reactionless based on the reconfiguration concept is put forward, which can address the mentioned problem. With the mechanism reconfiguration, the control system needs to be adapted thereafter. One way to address control system adaptation is by applying the “divide and conquer” methodology. It entails modularizing the functionalities: breaking up the control functions into small functional modules, and from those modules assembling the control system according to the changing needs of the mechanism.

## 1. Introduction

Robotic systems can be generally divided into parallel manipulators and serial manipulators. A parallel manipulator, also sometimes called a parallel mechanism, is a type of machine where the movable platform is joined to its base through at least two limbs and the whole system forms a closed loop. The counterpart of a parallel manipulator is a serial manipulator. A serial manipulator consists of links that are connected by joints in a serial fashion and the system is an open loop structure. Parallel manipulators have been widely used in numerous areas, such as conduct manufacturing machining [1], medical devices [2], and sensor applications [3], due to their performance characteristics. The most well-known parallel manipulator is the Steward platform. The movable/upper platform joins to its base through six actuated limbs, and the manipulator has six degrees of freedom, i.e., three translational movements along the three axes, and three rotational motions around the three axes. Serial manipulators can be used in applications where high accuracy is not required and a large workspace is desired, for example, serial manipulators used in entertainment, serving senior people, excavating, assist rough machining, etc. The advantages of the parallel manipulators compared to the serial manipulators are that parallel manipulators possess high stiffness, high rigidity, high accuracy, high speed and acceleration, and no cumulative joint/link error, due to the parallel structure arrangement. The advantages of serial ones are that they can reach larger workspace comparing to parallel ones.

Many other kinds of parallel manipulators having been designed and developed during the past decades, all with purpose of further improving their performance [4,5,6,7], e.g., stiffness, workspace, positioning accuracy, acceleration, and dynamic characteristics. In reference [8], a 3-DOF tripod manipulator was proposed by Zhang, where a passive link is added to the system with the aim of increasing the stiffness of the robotic system and eliminating unexpected movements. In reference [9], a TAU hybrid manipulator was proposed. The advantage of this type of manipulator is that it combines the features of serial manipulators and parallel manipulators, which increases its end-effector workspace and its stiffness still remains high. 

When parallel manipulators move, because the position of the center of mass (CoM) and also the angular momentum are changing, there are vibrations within the system, which can degrade the accuracy performance. How to reduce those vibrations has become a common desire. To reduce or ideally eliminate the system vibrations, one can apply dynamic balance methods. The purpose of dynamic balancing is to make the CoM fixed and the angular momentum of the system constant. In most of the cases, for ease of analysis, one usually makes the system’s linear and angular momentum equal to zero. In some cases where for applications (i.e., parallel robots adopted as machine tools, surgical devices or sensors) that are commonly characterized by low velocities and accelerations, static balancing may provide a suitable approach [10,11], even in cases where counterweights are installed [12,13,14]. In addition, it is advised that making the CoM invariant through force balancing (which is a necessary condition for achieving complete dynamic balancing) may still result in a non-zero shaking force (and shaking moment) due to elastodynamic phenomena [15,16,17]. Elastodynamic phenomena is out of the scope of this study, and will be addressed separately in future work. Here we mainly consider the vibration that is caused by the dynamic unbalanced conditions. Normally, dynamic balancing is accomplished through employing counter-masses or counter-rotation approaches [18,19,20]. The potential issue is that more weight and inertia are added to the system. Here it is proposed to accomplish dynamic balance based on designing naturally dynamic balanced mechanisms rather than employing counterweights or damping, for example, based on the developed reconfiguration method. Once a dynamic balanced single limb is developed by the reconfiguration method (decomposition), the limbs are synthesized to construct parallel manipulators (integration); i.e., the concept of decomposition first and integration later.

Controlling a robotic system to act in a designated way is a challenging task because robotic manipulator mechanisms are extremely nonlinear. For robotic manipulators, the coefficients of the dynamic equations are functions of the joint variables and also a function of the payload mass which may be unknown or change throughout the task. When the manipulator moves, the joint variables are changing, which will cause the robotic manipulator’s dynamic equation to change throughout a given task. In order to obtain a high degree of accuracy and repeatability in the manipulator performance, it is imperative that a more advanced control technique (e.g., MRAC) be used to account for the changes in the dynamic characteristics of the manipulator. Traditional control approaches make mechanism-uncoupled linear subsystems, and they are able to supply decent performance at low speeds. However, for high speed actions, they are not sufficient anymore. The use of the PID controller for control does not guarantee optimal control of the system or system stability. For the purpose of resolving the above issue, one can resort to (model reference) adaptive control as it is the most prevalent and well-established method. The MRAC method was first introduced by Whitaker et al. in 1958 [21], when they considered adaptive aircraft flight control systems, employing a reference model to determine error signals between the actual and desired behavior. These error signals were utilized to alter the controller parameters to reach ideal behavior in spite of uncertainties and varying system dynamics. The MRAC was later developed further [22,23,24,25]. Dubowsky [26] was the first to apply the MRAC to a robotic manipulator. A steepest-descent method was used for updating the feedback gains, after which the hyper-stability method [27] was utilized here and applied to a serial robotic arm.

The organization of this paper is as follows: Section 2 presents a novel 3-DOF hybrid manipulator synthesis design; dynamic balancing through reconfiguration and integration design are presented in Section 3; Section 4 describes the design for a hybrid controller for serial robotic manipulators in order to further improve the accuracy and joint convergence speed performance. Conclusions are presented in Section 5.

## 2. Synthesis Design

Parallel mechanism synthesis design is an important exercise for parallel manipulator development and applications. The type of synthesis of parallel manipulators is and will still be a main issue for many scholars and its related applications. Type synthesis can simply be put this way: using diverse approaches to integrate the merits of serial mechanisms and parallel mechanisms to design new application-orientated mechanisms. Furthermore, if one wants to design a new set of parallel mechanism, one cannot stick to the traditional joints, i.e., prismatic joints, revolute joints, universal joints and spherical joints. One can use, for example, parallelogram (Pa joints) or pure-translational universal joints (U* joints; U* joints can also be seen as a parallelogram) or the double parallelogram (Pa2 joints) as a limb rather than sticking to traditional fixed length limb, because the parallelogram formation has better stiffness performance compared to ordinary limbs. Obviously, parallelograms have double linkages, which can distribute the loads whereas the normal leg just has one leg, so the stiffness for the parallel robotics that are based on the parallelogram formation is better than that of the non-parallelogram form ones. Furthermore, the rotational capability will be enhanced [28] if one employs parallelograms. Until now, numerous methods have been proposed to give general instructions for the purpose of creating new sets of parallel mechanisms to improve parallel manipulators’ performances. The most common way to design a parallel mechanism is the one that based on the Chebychev-Grübler-Kutzbach template and then enumerate all the possibilities, which is a cumbersome task. In reference [29], the systematic enumeration method for spatial parallel manipulators which is based on the concept some of the ideal manipulators’ functional requirements are transformed into structural characteristics is proposed. First, one has the following formula:

(1) $$\begin{array}{cc} \sum_{k}^{m}C_{k}=7F-6 & \left(F\leq C_{k}\leq 6\right) \end{array}$$

where *C_k_* is the connectivity of each limb, and *F* is the end-effector’s number of degrees of freedom. According to Equation (1), and for the 2- to 6-DOF of parallel manipulators, we can list a table that illustrates all the possible solutions. Here it is assumed that the number of limbs equals the DOF of the manipulator. 

Each connectivity listing given in the Table 1 represents a family of parallel manipulators for which the limb number is the same as the degrees of freedom of parallel mechanism, and the total number of joint DOF in each limb/leg equals to the value of *C_k_*.

Let us consider a case study: say one wants to invent a 3-DOF pure translational parallel manipulator. This involves the following steps:

Step 1 here we limit our research to those manipulators having three identical limb structures, so the “5 5 5” option in Table 1 is the only solution.
Step 2 a combinatorial analysis can yield the following four types of limb configuration, as shown in Table 2.
Step 3 it is preferable to have a base-connected R or P joint, and an intermediate P joint for actuation, so some of the ones listed in Table 2 can be eliminated. Further, since the S joint is not able to constrain the moving platform rotation due to the fact that we need to invent a pure translation manipulator, the entire “201” type will be eliminated. Then the rest will be the feasible manipulators.

In reference [30], the theory of groups of displacements is used to develop some new architecture of 4-DOF 3T1R parallel mechanisms by resorting to the parallelograms. The parallelogram structure is treated as a “motion engine”, and by combining “motion engines”, a new set of parallel mechanisms can be generated; in a similar way as illustrated in [31], two of the same kinematic chains which are used as “Schonflies-Motion Engines” are developed for the parallel mechanism, and they employed the parallelograms as well. The general function (Gf) set theory was put forward recently to synthesize design parallel mechanisms [32], and our new proposed manipulator is based on this method. Now let’s design a 3-DOF (two rotational DOF around the X and Y axes and one translational DOF along the Z axis) parallel manipulator based on the Gf set theory. According to the general function set theory, it has two classes. The first class is $G_{F}^{I}(\begin{array}{ccc} T_{a} & 0 & 0 \end{array};\begin{array}{ccc} R_{\alpha } & R_{\beta } & 0 \end{array})$, and the second class is $G_{F}^{II}(\begin{array}{ccc} R_{\alpha } & R_{\beta } & 0 \end{array};\begin{array}{ccc} T_{a} & 0 & 0 \end{array})$. 

For the first class: first of all, we need to determine how many linkages, how many active linkages, how many passive linkages and how many actuators on the *i*th active linkage should be employed by using the following Equations (2) to (6) based on the end-effector characteristics:

(2) $$D_{e}-\sum_{i=1}^{g}a_{i}=0$$

(3) $$L=D_{e}-\sum_{i=1}^{g}\left(a_{i}-1\right)+l$$

(4) L=g+l

(5) $$a_{i}\leq D_{e}$$

(6) $$g\leq D_{e}$$

where *D_e_* is the dimension of the end-effector characteristics, *l* is the number of passive legs, *a_i_* is the number of actuators on the *i*th actuated leg, *g* is the number of actuated legs, and *L* is the number of legs.

Secondly, based on the intersection algorithms, we need to produce the categories of composition of the characteristic of the EE for the class $G_{F}^{I}(\begin{array}{ccc} T_{a} & 0 & 0 \end{array};\begin{array}{ccc} R_{\alpha } & R_{\beta } & 0 \end{array})$.

Thirdly, according to the types of composition of the characteristic of the EE we just obtained in the step two, we can find the required kinematic legs/limbs with required characteristic of the EE, i.e., all the possibilities kinematic chains;

Finally, the particular ideal parallel mechanism can be synthesized through gathering the kinematic limbs. 

For the second class $G_{F}^{II}(\begin{array}{ccc} R_{\alpha } & R_{\beta } & 0 \end{array};\begin{array}{ccc} T_{a} & 0 & 0 \end{array})$, the procedure is the same with the first class except in the second step that we need to obtain the types of composition of the characteristic of the EE for the class $G_{F}^{II}(\begin{array}{ccc} R_{\alpha } & R_{\beta } & 0 \end{array};\begin{array}{ccc} T_{a} & 0 & 0 \end{array})$.

**Figure 1:**
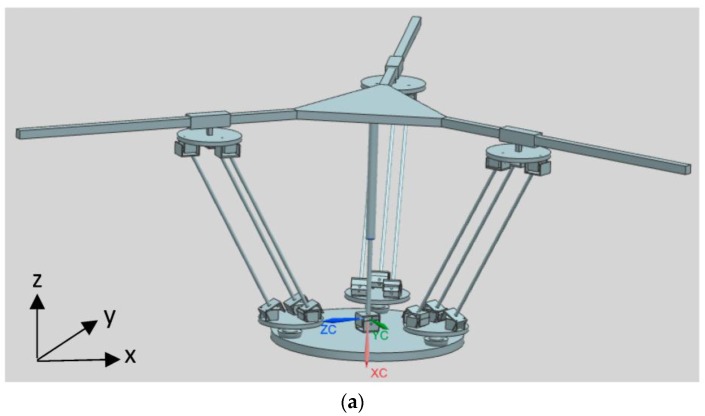

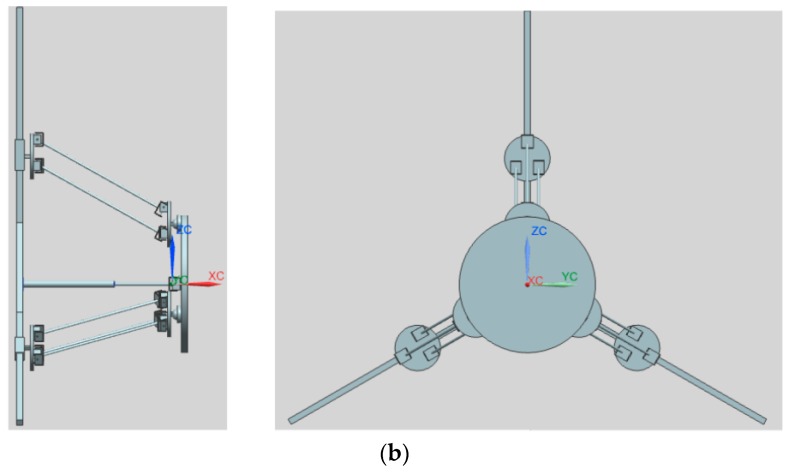
(**a**) Isometric view a 3PU*S-PU parallel manipulator (**b**). Side and top views of a 3PU*S-PU parallel manipulator.

Based on the above procedure, a parallel/hybrid 3PU*S-PU mechanism as illustrated in Figure 1 is derived. In fact there are multiple 3-DOF parallel mechanisms that can be derived based on the general function set theory [33], but some of them are not useful at all. For example, among them, one is the 3PU*S-(CR)_o_ parallel manipulator and another is the 3PU*S-(RC)_o_ manipulator, but these two parallel mechanisms have a rotational axis that has a 90° angle with respect to the moving platform, i.e., one of the rotational movement is around the Z axis, which is not what the authors wanted−what one wanted is two rotational movements around the X and Y axes, respectively, i.e., the angle of the two rotational axes with respect to the moving platform is zero. In reference [34], a hybrid robotic manipulator was proposed to be used as the neck of a mine rescue robot. This robot was inspired by a bio-structure, and the distinct attribute of that manipulator is that a passive leg which is in the form of prismatic-universal structure was put in the middle inside the system so that it can constrain the whole structure to be 3-DOF, i.e., two rotational movements around the X and Y axes and one translational movement along the Z axis.

Inspired by the design in reference [34], the middle passive limb was switched from the original type to the prismatic-universal structure pattern, this passive limb equipped with a universal joint (it is fixed at the moving platform center) and a prismatic joint (it is connected to the base), by this way the mechanism has the three desired DOF, i.e., two rotational movements around the X and Y axes, and one translational movement along the Z axis.

The novelty of the new proposed 3PU*S-PU manipulator can be summarized as follows: firstly, by employing the U* joint, the stiffness of this parallel manipulator can be greatly improved since the translational universal joint (U* joint) is a type of the parallelogram family of joints. The translational universal joint has two major bits, the first part is the two platforms, the other one is the three linkages that join those two platforms via U joints, from which one can see that payloads on the platform can be spread along those three linkages, therefore the stiffness of the parallel manipulator using U* joints is higher than that using normal legs. Please further note that there are actually two purposes of using U* joints, one is to enhance the parallel mechanism’s stiffness performance, and another is to increase the titling angle or rotational capability of the end-effector because the translational universal joint’s performance bears resemblance to a parallelogram, and the parallelogram can enlarge the parallel mechanism’s rotational capability [28]. Secondly, by altering the middle passive linkage’s pattern, i.e., changing the degrees of freedom of the passive linkage, the whole parallel mechanism will be able to achieve reconfiguration, for example, if one removes the passive linkage, the three degrees of freedom mechanism will become six degrees of freedom.

Here the manipulator the authors proposed only has 3-DOF, so the second purpose of the U* joint that we just mentioned is deactivated by the central passive prismatic-universal linkage, because in machine tool design normally we just need three degrees of freedom, i.e., one translation along Z axis and two rotations about X and Y axes. If one wants to design manipulator systems with more than three degrees of freedom on the basis of the hybrid mechanism we propose, one can remove the middle passive leg, then the system will come to be a 6-DOF mechanism. How the 6-DOF manipulator can be controlled will be addressed in future work. By altering the central passive linkage’s pattern, i.e., changing the degrees of freedom of the central passive linkage, the whole parallel mechanism can achieve reconfiguration.

## 3. Dynamic Balancing Design

When mechanisms and parallel manipulators move, the position of the center of mass is changing and the angular momentum is also changing, therefore vibration is produced inside the system. The purpose of dynamic balancing is to make the CoM fixed and the angular momentum unchanging. In most cases, for ease of analysis, one usually make the system’s linear and angular momentum equal to zero. Normally, the existing shaking force and shaking moment that the whole system produces can be dynamically balanced by adding supplementary components. However, the potential issue that more weight and inertia will be included inside the system, which can make the system heavy and produce higher inertia effects, appears. Here it is proposed to achieve dynamic balance through a reconfiguration method, and the mass relation index is proposed as a basis to further derive new reactionless mechanisms. Furthermore, after a single dynamic balanced limb is created, those limbs can be assembled together to construct the entire parallel structure. 

**Figure 2:**
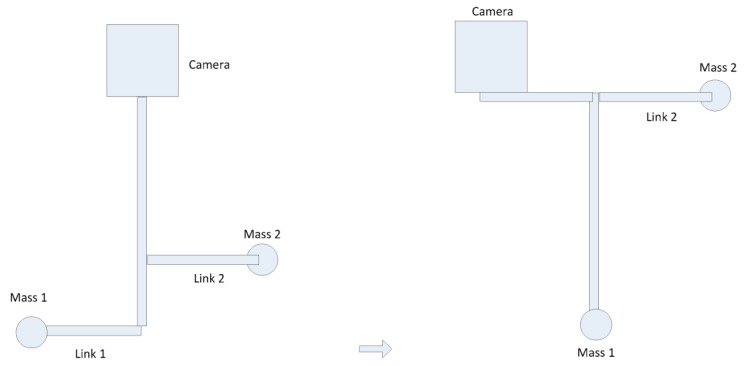
SteadiCam operation.

Here the authors suggest that one can accomplish dynamic balancing conditions based on employing naturally dynamically balanced mechanisms rather than resorting to the old counterweights approaches. For instance, one can accomplish reactionless conditions based on the reconfiguration concept. Here a new approach is put forward, i.e., balancing via reconfiguration, which can maintain the system weight and inertia and not make the system heavier. For a SteadiCam system, as shown in Figure 2, counter-weights are employed in order to make the system force balanced, and by altering the mass relations, make the system dynamically balanced. This is a mass relations concept. There are two linkages at the bottom of the system, and those two linkages can function as the counter-mass, so the system is force balanced. If one whirls the SteadiCam system, the system is dynamic balanced as well. If the link 2 is relocated somewhere towards the up direction, as illustrated in Figure 2, the system is still force balanced, but the dynamic balance condition will be lost. Therefore the problem one faces is how to reposition or reconfigure the layout of the system in order to recover the reactionless condition.

Let us consider the situation where one shifts link 2 to a different position, i.e., we relocate it to the top. It is clear that if the reactionless condition needs to be brought back, one has to shift the camera in a counter-clockwise direction, as shown in the Figure 2, and the same goes for mass 1. Therefore one has the same scenario, the only difference is that there are two weights in the top instead of one and one weight in the bottom instead of two. Put it in another way, if the linkage 2 is shifted counter-clockwise, one also has to shift the camera counter-clockwise, and the same goes for mass 1. In this way, reactionless conditions can be regained. The whole process concerns the mass relations, so as long as one has the required mass relationships, one can have reactionless conditions. What dominates here is the correlation of those three masses.

**Figure 3:**
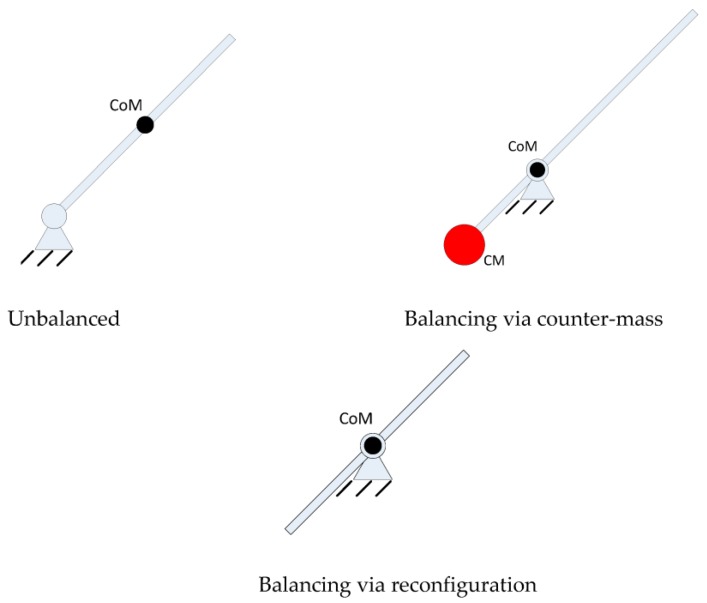
Concept of balancing through reconfiguration.

Inspired by the above design, here dynamic balancing based on the reconfiguration notion is put forward. Taking the following case as an example, one can employ ac screw linkage and the linkage is able to shift, in this way the linkage’s center of mass can be shifted to the position where the revolute joint resides, and it is therefore balanced. Through this approach, one does not employ a counter-mass but via reconfiguring the system by shifting the linkage, which does not make the system heavier. The following Figure 3 illustrates the notion of balancing based on reconfiguration.

The position for the link’s CoM relative to the coordinate system (x, y) is expressed as:

(7) $$c_{1}=\left[\begin{array}{l} x_{1}\\ y_{1} \end{array}\right]=\left[\begin{array}{l} d_{1}\;\text{cos}\;\beta_{1}\\ d_{1}\;\text{sin}\;\beta_{1} \end{array}\right]$$

where *d*_1_ is the distance from the CoM of the link to the revolute joint, *β*_1_ is the rotation angle of the link with respect to the x axis. The origin of the coordinate frame (x, y) coincides with the revolute joint, the x axis horizontally points towards right, and the y axis vertically points up. The linear momentum of the linkage is therefore:

(8) $$m_{1}\overset{⦁}{c_{1}}=m_{1}\left[\begin{array}{l} \overset{•}{x_{1}}\\ \overset{•}{y_{1}} \end{array}\right]=m_{1}\left[\begin{array}{l} -d_{1}\;\text{sin}\;\beta_{1}\\ d_{1}\;\text{cos}\;\beta_{1} \end{array}\right]\overset{•}{\beta_{1}}$$

where *m*_1_ is the mass of the link. For the aim of getting force balancing conditions, the linear momentum has to be set constant. By observing the above equation, and since the mass cannot be set to zero, the only way to make it a constant is to set *d*_1_ to zero, which means the CoM of the linkage is set to the revolute joint:

(9) $$m_{1}d_{1}=0\to d_{1}=0$$

**Figure 4:**
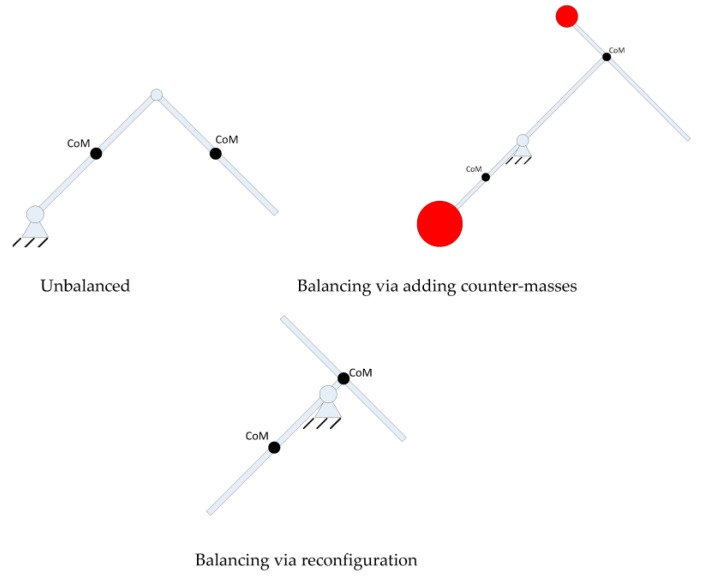
Balancing of a 2-DOF serial link via reconfiguration.

Employing this concept and applying it to two-bar three-bar, four-bar and other mechanisms, new balanced two-bar three-bar, four-bar and other mechanisms can be derived. The objective of employing counter-weights is to shift the center of mass to a fixed spot, so the concern one faces is whether it is possible to not employ counter-weights to accomplish the same aim. The link can be reconfigured so that center of mass is shifted to a fixed spot. Now for the two link scenario, we have the following result, as shown in Figure 4.

The position for the link 2’s CoM relative to the coordinate system (x, y) is expressed as:

(10) $$c_{2}=\left[\begin{array}{l} x_{2}\\ y_{2} \end{array}\right]=\left[\begin{array}{l} l_{1}\;\text{cos}\;\beta_{1}+d_{2}\;\text{cos}\;\beta_{2}\\ l_{1}\;\text{sin}\;\beta_{1}+d_{2}\;\text{sin}\;\beta_{2} \end{array}\right]$$

where the mass and length of link 1 is denoted as *m*_1_ and *l*_1_, respectively, and the mass of link 2 is denoted as *m*_2_ and *l*_2_, respectively. *d*_2_ represents the distance from the center of mass of linkage 2 to revolute joint 2, and *β*_2_ is the rotation angle of link 2 relative to the x axis. The linear momentum of the linkage is therefore:

(11) $$\begin{array}{cc} m_{1}\overset{•}{c_{1}}+m_{2}\overset{•}{c_{2}} & =m_{1}\left[\begin{array}{l} \overset{•}{x_{1}}\\ \overset{•}{y_{1}} \end{array}\right]+m_{2}\left[\begin{array}{l} \overset{•}{x_{2}}\\ \overset{•}{y_{2}} \end{array}\right]\\ & =m_{1}\left[\begin{array}{l} -d_{1}\;\text{sin}\;\beta_{1}\\ d_{1}\;\text{cos}\;\beta_{1} \end{array}\right]\overset{•}{\beta_{1}}+m_{2}\left[\begin{array}{l} -l_{1}\;\text{sin}\;\beta_{1}\overset{•}{\beta_{1}}-d_{2}\;\text{sin}\;\beta_{2}\overset{•}{\beta_{2}}\\ l_{1}\;\text{cos}\;\beta_{1}\overset{•}{\beta_{1}}+d_{2}\;\text{cos}\;\beta_{2}\overset{•}{\beta_{2}} \end{array}\right]\\ & =\left[\begin{array}{l} m_{1}(-d_{1}\;\text{sin}\;\beta_{1})+(-m_{2}l_{1}\;\text{sin}\;\beta_{1})\\ m_{1}(d_{1}\;\text{cos}\;\beta_{1})+(m_{2}l_{1}\;\text{cos}\;\beta_{1}) \end{array}\right]\overset{•}{\beta_{1}}+\left[\begin{array}{l} -m_{2}d_{2}\;\text{sin}\;\beta_{2}\\ m_{2}d_{2}\;\text{cos}\;\beta_{2} \end{array}\right]\overset{•}{\beta_{2}} \end{array}$$

For the aim of getting the force balancing conditions, the linear momentum has to be constant. From observation of the above equation, in order to satisfy the above condition, the following force balancing conditions are therefore obtained:

(12) $$\begin{array}{l} m_{1}d_{1}+m_{2}l_{1}=0\\ m_{2}d_{2}=0 \end{array}\}\to \left\{ \begin{array}{l} d_{1}=-\frac{m_{2}l_{1}}{m_{1}}\\ d_{2}=0 \end{array}\right.$$

From the above equation, the center of mass of the linkage 2 is set to revolute joint 2 and the CoM of link 1 is at the point where the distance to revolute joint 1 is $m_{2}l_{1}/m_{1}$.

**Figure 5:**
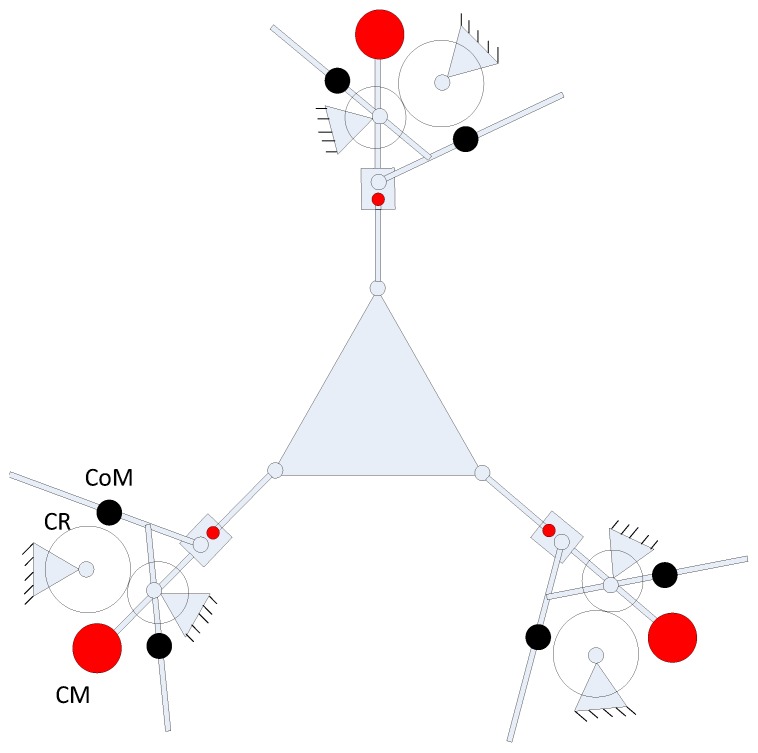
Dynamically balanced 3RPR planar parallel robotic system.

Based on the previous analysis, one can see that force balancing based on the reconfiguration notion will not include an extra counter-mass, and on the other hand, if balancing is accomplished by resorting to adding an additional counter-mass, the entire system be heavier. After applying this concept to the crank-slider mechanism, the crank-slider system which is balanced based on the reconfiguration notion is used as a Scott-Russell mechanism in place of the conventional version type [18] and we join the crank-slider system to every single limb of the 3-RPR parallel mechanism, as illustrated in Figure 5. In order to achieve complete dynamic balancing, three counter-rotations have been added within the system, as illustrated in Figure 5, where CR stands for counter-rotation, the red solid circle represents a counter-mass, and the black solid one stands for the center of mass.

The moving platform mass can be replaced by three point masses placed at three attachment points of the moving platform and the three legs. The three point masses are represented by *m_pm_*_1_, *m_pm_*_2_, and *m_pm_*_3_. If one satisfies the following, then the above condition can be obtained:

(13) $$\left\{ \begin{array}{l} m_{pmi}=\frac{m_{mf}}{3}\\ I_{pm}=3m_{pmi}{R_{mf}}^{2} \end{array}\right.$$

where *m_mf_* and *R_mf_* are the mass and radius of the moving platform, respectively. This replacement of the moving platform allows one to analyze the shaking force balancing and shaking moment balancing of each limb of the robotic system. Through making the linear and angular momentum equal to 0, the shaking force and shaking moment are able to be balanced provided:

(14) $$m_{bm1}=\frac{-m_{l1}r_{1}}{r_{bm1}}$$

(15) $$m_{bm2}=-\frac{m_{l2}r_{2}+m_{pmi}}{r_{bm2}}$$

(16) $$0=I_{l4}+(m_{l4}{r_{4}}^{2}+m_{l3}){l_{4}}^{2}-I_{l3}-m_{l3}{r_{3}}^{2}{l_{4}}^{2}$$

(17) $$\begin{array}{cc} I_{br} & =(m_{l4}{r_{4}}^{2}+m_{l3}{\left(1-r_{3}\right)}^{2}){l_{4}}^{2}+I_{l4}+I_{l3}+I_{l2}+I_{l1}\\ & +(m_{l2}{r_{2}}^{2}+m_{bm2}{r_{bm2}}^{2}+m_{l1}{r_{1}}^{2}+m_{bm1}{r_{bm1}}^{2}+m_{pmi}){l_{2}}^{2} \end{array}$$

where *m_bmi_* is the mass of the counterweight *i*. The mass and axial moment of inertia of link *i* are denoted as *m_li_* and *I_li_*, respectively. *r_i_* and *r_bmi_* are dimensionless coefficients. The counter-rotations’ axial moment of inertia is denoted as *I_br_*. 

It can be seen that by employing the reactionless via reconfiguration to a crank-slider system to function as a Scott-Russell system, no extra counter-mass is included inside the system. However, if one continues to use the conventional version type, two extra counter-masses must be included in the Scott-Russell system, which will increase the overall mass and inertia. By employing the reconfiguration approach as an alternative to adding counter-masses, it is also possible to make the 4-bar linkage equipped with an Assur group [13] achieve reactionless condition, and employ those reconfigured based reactionless 4-bar linkage equipped with an Assur group to synthesize the entire parallel robotic system, put it another way, one can decompose the system first, and after that integrate the system. The above shows the concept of dynamic balancing based on the reconfiguration approach, as an alternative to adding counter-masses, the objective of which is to shift the center of mass, so one is able to resort to the reconfiguration technique to accomplish the same aim.

### Testing

**Figure 6:**
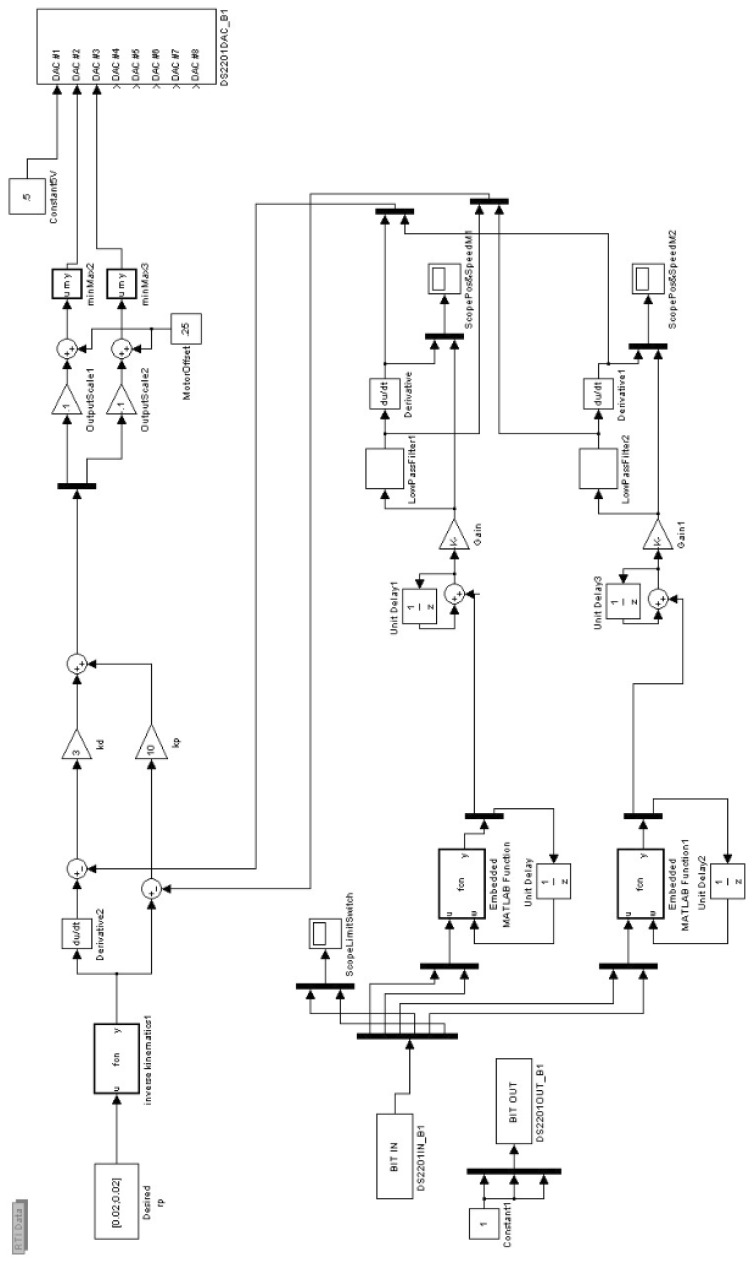
Simulink model.

**Figure 7:**
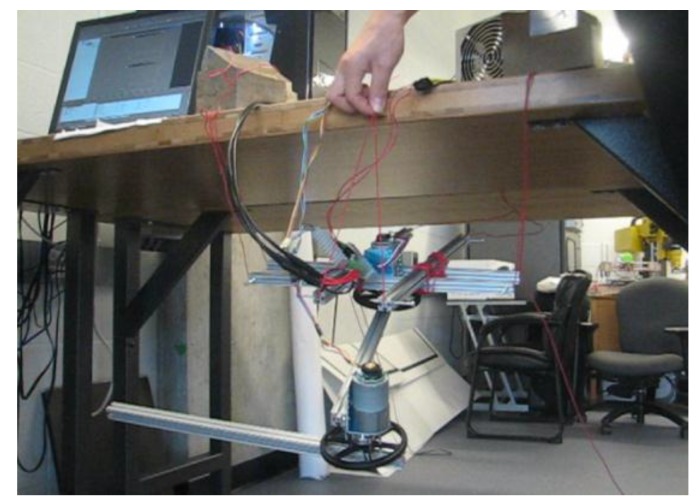
Unbalanced 2-DOF link case.

**Figure 8:**
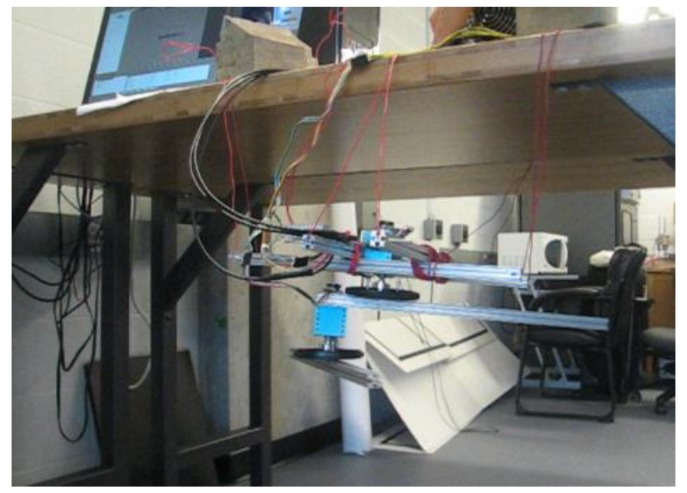
Balanced 2-DOF link case.

Testing was conducted using Simulink and dSpace. Here, a 2-DOF link manipulator is set up and built as an illustration. The robot is suspended by wires in the air so any unbalancing phenomena can be easily observed. The Simulink model is shown in Figure 6. For the unbalanced 2-DOF link manipulator, when the manipulator moves from one position to another, the system will swing and vibrate, which can be observed in real time, as illustrated in Figure 7. For the balanced 2-DOF link case, when the manipulator moves from one position to another, the system will remain steady, which can also be observed in real time, as shown in Figure 8.

## 4. Adaptive Control Design 

Control of a serial mechanism can be categorized as joint control and end-effector operational control. Most robotic industries use a PID controller to control each joint of robotic manipulators. The problem is that it cannot make up for any load changes. When the robotic arm’s end-effector grasps different payload masses, the output of the joint motion varies, which decreases the end-effector positioning accuracy of the robotic arm system.

**Figure 9:**
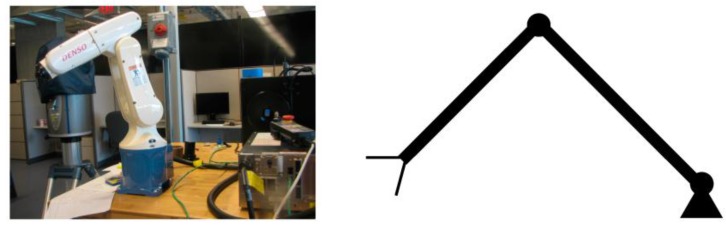
Two-link manipulator.

**Figure 10:**
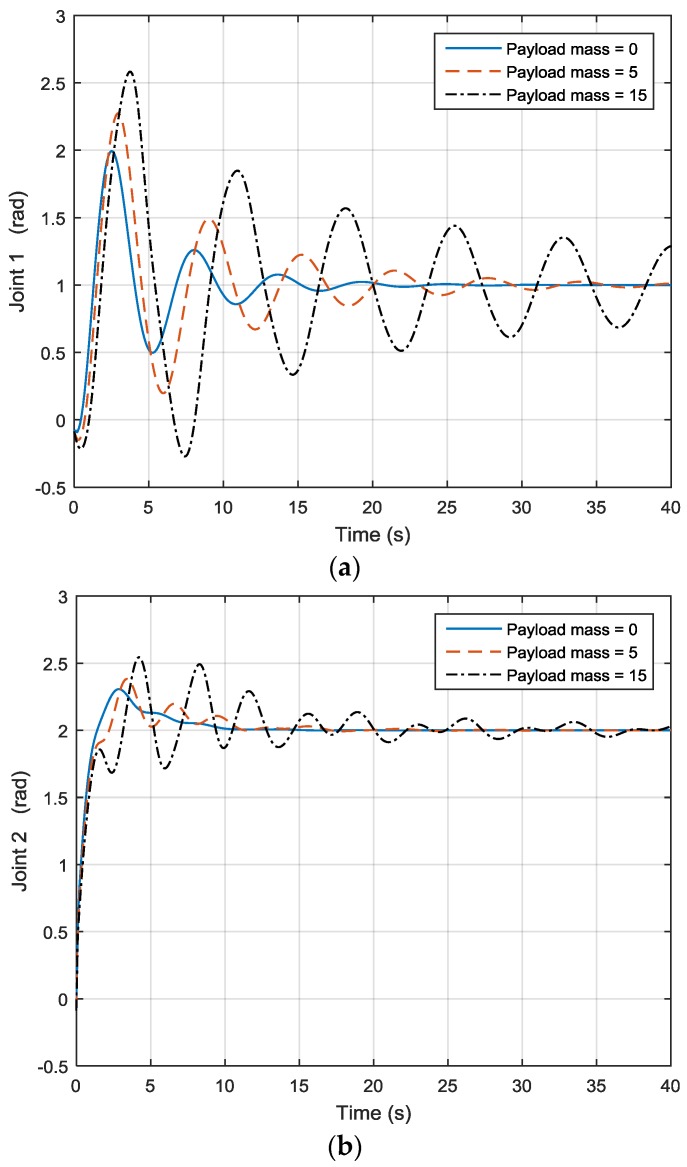
(**a**) Joint 1 output; (**b**) Joint 2 output.

Here a two-DOF link manipulator (as shown in Figure 9) will be used as an example. After applying different payload masses, joint 1 motion output is illustrated in Figure 10a while joint 2 motion output is shown in Figure 10b. For joint 1, when the payload is 0, the motion is quite steady, but when the payload increases to 5 and 15, one can see that joint 1 motion is no longer the same, as shown in Figure 10a, and the simulation also shows that the joint output increases and decreases. The same applies to joint 2, as seen in Figure 10b. This has led to the use of adaptive control, especially model reference adaptive control (MRAC). Dubowsky [26] was the first to apply the MRAC concept to a robotic manipulator. The author employed a linear time-invariant differential formulation to be the reference model for each joint of the robot. The robotic system was maneuvered through altering the feedback gains to follow the model.

A steepest-descent approach was employed to update the feedback gains, after which Horowitz applied the hyper-stability method and developed an adaptive algorithm [34] for a serial fashion robot arm to which a nonlinear term in its dynamic equation was compensated and the dynamic interaction among the joints was decoupled. The adaptive method proposed by Horowitz in [34] differs from Dubowsky’s technique [26]. 

Two major distinctions can be summarized as follows: first, in Horowitz’s method, the entire control system consists of a MRAC inner loop system and a position and velocity feedback outer loop system, while the control architecture for Dubowsky’s approach is completely based on MRAC; second, for Dubowsky’s approach, the existing coupling between each joint and nonlinear provisions in the dynamic equations are not considered, on the contrary, they are included for the Horowitz approach. The drawback of Horowitz’s approach is that the matrices M and N are assumed constant, which means one cannot employ the normal Lagrange method to obtain the dynamic equation of robotic manipulators to control the robotic arms. In reference [27], Horowitz applied the Gibbs-Appell formulation for dynamic modelling of robotic manipulators to meet the requirement that inertia matrix and non-linear provision in the system dynamic equation are constant. 

An improved version of the method was later proposed in Sadegh [35]. The assumption that the inertia matrix and nonlinear provision are constant in the process of adaptation can be removed by altering the control rule and parameter adaptation rule. It was proved that, through altering the control rule (i.e., by forming the Coriolis and centripetal compensation control system a bilinear expression of the joint and model reference velocities, as an alternative to a quadratic expression of the joint velocities) and through altering the parameter adaptation rule (i.e., by decomposing the nonlinear parameters in the dynamic equation of the robotic system to the multiplication of two terms: one term is a constant unknown part, which has the masses, moments of inertia of the linkages, payload and linkage dimensions in it, and the other term is a known nonlinear expression of the robotic system structural dynamics), the assumption that the inertia matrix and nonlinear term are constant during adaptation is removed.

Based on the MRAC control and by integrating the PID control, a PID + MRAC hybrid controller is proposed for serial robotic manipulators. In Horowitz’s MRAC control system [27], the inertia matrix and non-linear provision M and N are treated as constants in the adaptation process. With respect to the one-link scenario, due to the fact that the M matrix is constant, there is no N matrix, and one is able to straightforwardly combine the PID and MRAC controllers to create the PID + MRAC control system. However, for the case of over 1-DOF linkage, one cannot repeat the above process as the dynamic equation’s inertia matrix and non-linear provision are not constant.

**Figure 11:**
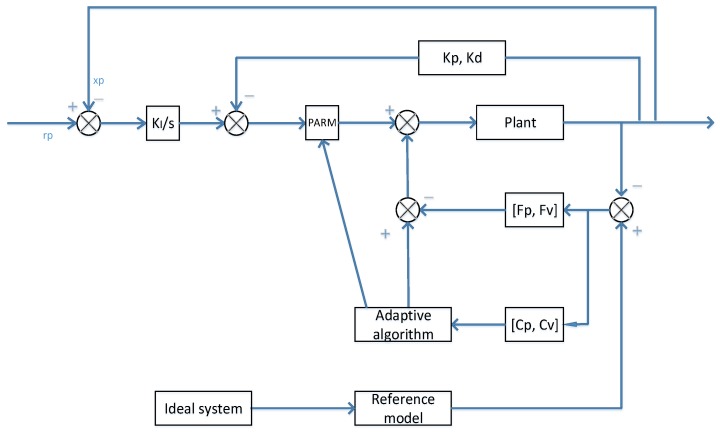
MRAC control system.

Regarding the MRAC controller, Figure 11 shows such a system. The plant’s output is going to be compared to the reference model output, whereby this action results in an error. The error then is going to be processed inside the adaptation section and the result will be provided as one of the input parts to the plant. Meanwhile, the plant’s output is going to be compared to the desired model rp and another error is subsequently generated. This error is processed by the integration action and the result of which will subtract the position and velocity that are processed by the Kp and Kd elements. This procedure is comparable to the PID control. The output from this procedure, multiplies the products from the adaptation block, adds the products from the adaptation block, is going to be the plant’s input. This action continues till the error between the plant’s output and the output from the reference model goes to zero. The ideal system is independent from the plant, so that the plant feedback values are not being utilized to influence the reference model input. The reference model input will be dealt with by its own output variables via the “seemingly PID controller”. The ideal system is not influenced with regards to the plant.

**Figure 12:**
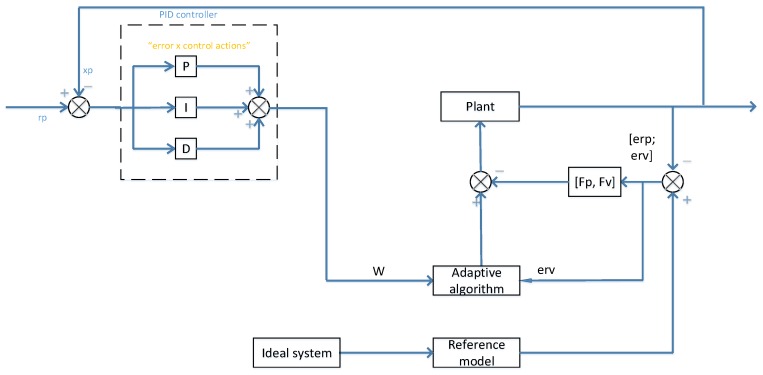
PID + MRAC hybrid control system for multi-DOF link.

Through integrating the PID control system and MRAC control system, a PID-MRAC hybrid control system can be obtained. In reference [27], the M and N matrices are treated as constant in the adaptation process. With respect to the simple one-linkage, due to the fact the M matrix is constant and there is no N matrix in the dynamic equation, the PID and MRAC control systems can be straightforwardly integrated to have the PID-MRAC control system. However, with respect to the case of over one linkage, one cannot repeat the above process as the dynamic equation’s inertia matrix and non-linear provision are not constant. Under the PID control, we need to employ the dynamic model which is derived based on the Lagrange approach, however, under the MRAC, one needs to employ the dynamic model which is derived based on the Gibbs-Appell dynamic formulation. Since these two are not well matched, the PID and MRAC cannot be integrated in this scenario. In reference [35], the author developed an improved MRAC system that is able to get rid of the situation where the M and N matrices being unchanging, in order to make the Lagrange based dynamic equation useable. Through employing Sadegh’s updated system, and through integrating the PID and MRAC controllers, a hybrid control system is put forward for multi-DOF linkage scenario, as shown in Figure 12. 

For the 2-DOF robotic manipulator, through employing the Lagrange technique, the dynamic formulation is illustrated as follows:

(18) $$\left[\begin{array}{l} \tau_{1}\\ \tau_{2} \end{array}\right]=M\overset{••}{\theta }+N=\left[\begin{array}{cc} m_{11} & m_{12}\\ m_{12} & m_{22} \end{array}\right]\left[\begin{array}{l} \overset{••}{\theta_{1}}\\ \overset{••}{\theta_{2}} \end{array}\right]+\left[\begin{array}{l} n_{11}\\ n_{21} \end{array}\right]$$

where $m_{11}=(m_{1}+m_{2}){l_{1}}^{2}+m_{2}{l_{2}}^{2}+2m_{2}l_{1}l_{2}\;\text{cos}\;\theta_{2}$, $m_{12}=m_{2}{l_{2}}^{2}+m_{2}l_{1}l_{2}\;\text{cos}\;\theta_{2}$, $m_{22}=m_{2}{l_{2}}^{2}$, $n_{21}=m_{2}l_{1}l_{2}\;\text{sin}\;\theta_{2}{\overset{•}{\theta_{1}}}^{2}$. $\theta_{1}$ and $\theta_{2}$ are the joint angles of joint 1 and 2, $l_{1}$ and $l_{2}$ are the length of links 1 and 2, and $m_{1}$ and $m_{2}$ are the masses of links 1 and 2. Through re-parametrization of the above equation,

(19) $$\left[\begin{array}{cc} (m_{1}+m_{2}){l_{1}}^{2}+m_{2}{l_{2}}^{2}+2m_{2}l_{1}l_{2}\;\text{cos}\;\theta_{2} & m_{2}{l_{2}}^{2}+m_{2}l_{1}l_{2}\;\text{cos}\;\theta_{2}\\ m_{2}{l_{2}}^{2}+m_{2}l_{1}l_{2}\;\text{cos}\;\theta_{2} & m_{2}{l_{2}}^{2} \end{array}\right]\left[\begin{array}{l} u_{1}\\ u_{2} \end{array}\right]+\left[\begin{array}{l} 2(-m_{2}l_{1}l_{2}\;\text{sin}\;\theta_{2})\overset{•}{\theta_{1}}\overset{•}{\theta_{2}}+(-m_{2}l_{1}l_{2}\;\text{sin}\;\theta_{2}){\overset{•}{\theta_{2}}}^{2}\\ m_{2}l_{1}l_{2}\;\text{sin}\;\theta_{2}{\overset{•}{\theta_{1}}}^{2} \end{array}\right]=W\cdot \left[\begin{array}{l} \Theta_{1}\\ \Theta_{2}\\ \Theta_{3} \end{array}\right]$$

By choosing $\Theta_{1}=(m_{1}+m_{2}){l_{1}}^{2}+m_{2}{l_{2}}^{2}$, $\Theta_{2}=m_{2}{l_{2}}^{2}$, and $\Theta_{3}=m_{2}l_{1}l_{2}$, one can obtain the *W* as follows:

(20) $$W=\left[\begin{array}{ccc} u_{1} & u_{2} & 2u_{1}\;\text{cos}\;\theta_{2}+u_{2}\;\text{cos}\;\theta_{2}-2{\overset{•}{\theta }}_{1}{\overset{•}{\theta }}_{2}\;\text{sin}\;\theta_{2}-{\overset{•}{\theta }}_{2}{\overset{•}{\theta }}_{2}\;\text{sin}\;\theta_{2}\\ 0 & u_{1}+u_{2} & u_{1}\;\text{cos}\;\theta_{2}+{\overset{•}{\theta }}_{1}{\overset{•}{\theta }}_{1}\;\text{sin}\;\theta_{2} \end{array}\right]$$

Since:

(21) $$M\overset{••}{\theta }+N=\tau =W\left[\begin{array}{l} \Theta_{1}\\ \Theta_{2}\\ \Theta_{3} \end{array}\right]-F_{v}\cdot erv-F_{p}\cdot erp$$

Therefore one has,

(22) $$\overset{••}{\theta }=(W\left[\begin{array}{l} \Theta_{1}\\ \Theta_{2}\\ \Theta_{3} \end{array}\right]-F_{v}\cdot erv-F_{p}\cdot erp-N)/M$$

**Figure 13:**
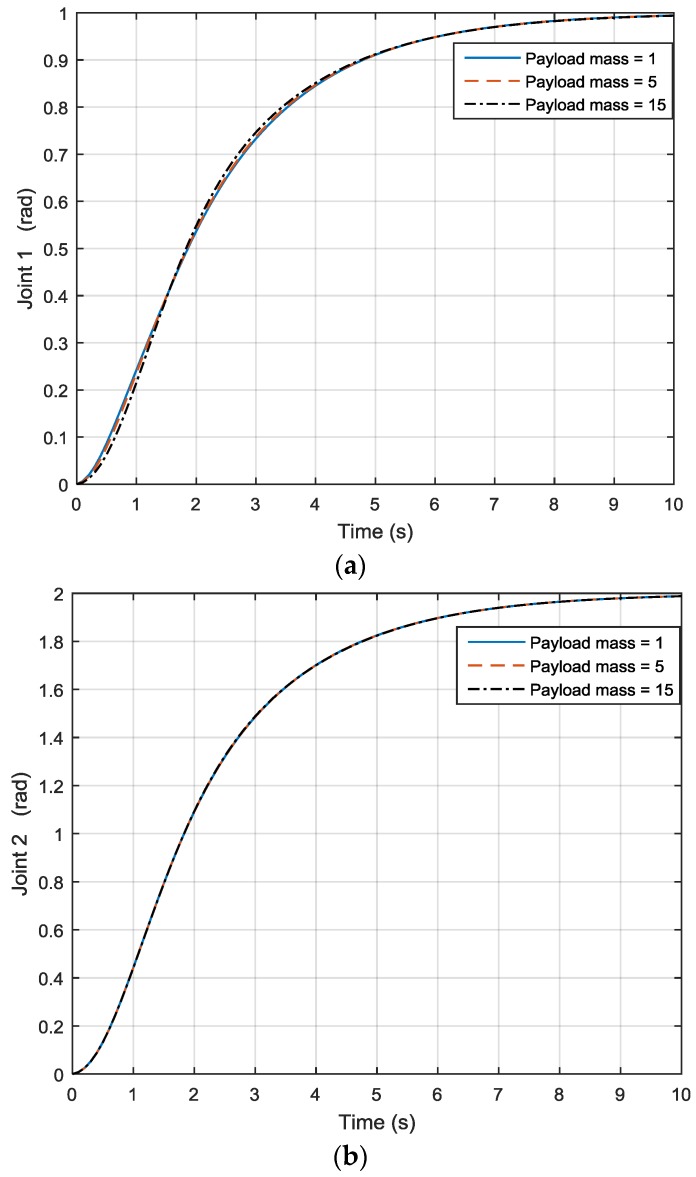
Outputs of (**a**) joint 1 and (**b**) joint 2 under hybrid control.

**Figure 14:**
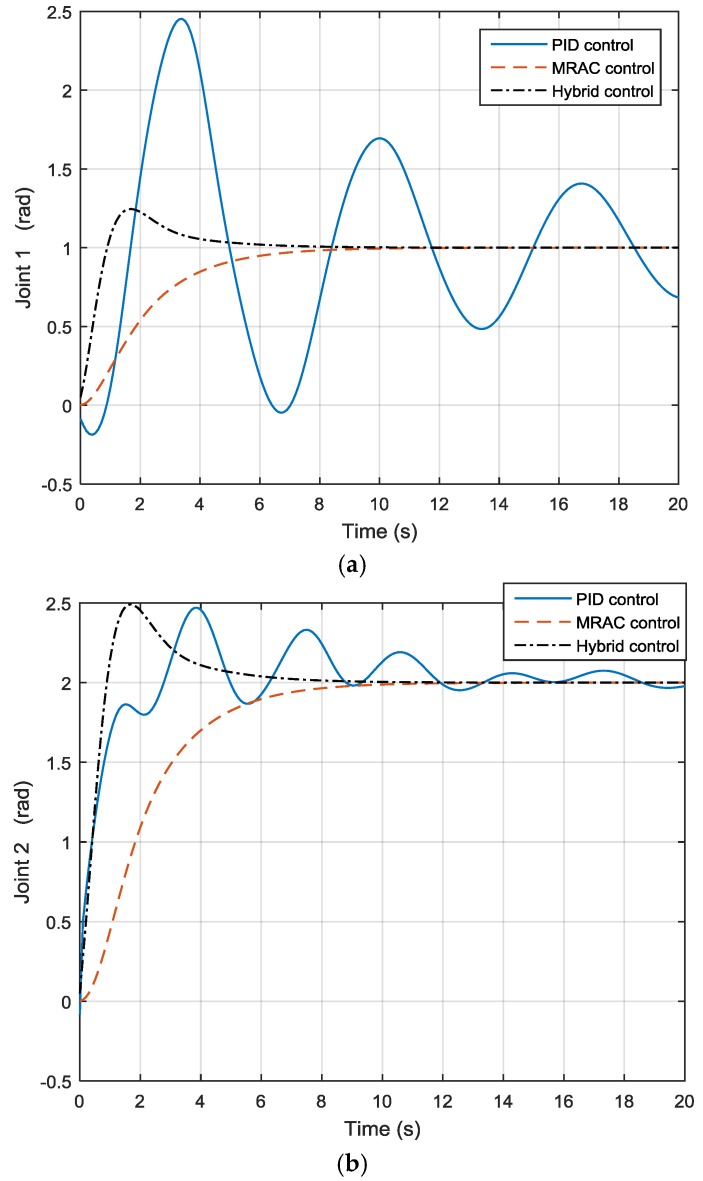
Outputs of (**a**) joint 1 and (**b**) joint 2 under three different controls.

As shown in the previous in Figure 10, after applying different payload masses and moving the robot from one position to another, when the payload is 0, joint 1 motion is quite steady, but when the payload increases to 5 and 15, joint 1 motion is no longer the same, and the joint output also increases and decreases. The same applies to joint 2. By using the hybrid approach, the three lines coincide with each other under different payload masses, as shown in Figure 13, so the payload mass variation effect has been compensated. Furthermore, as shown in Figure 14, one can see that the rate of convergence for the new hybrid control system is quicker than that of the MRAC control system, and the hybrid control and MRAC control systems both beat the PID control system.

## 5. Discussion and Conclusions

In this paper, performance improvement for robotic mechanisms is achieved by three different approaches, i.e., synthesis design, dynamic balancing design and adaptive control design. Firstly, for the synthesis design approach, a novel 3-DOF hybrid robotic mechanism 3PU*S-PU which is based on the general function set technique is proposed and analyzed. The novelty of this proposed new Gf set-based mechanism is that by altering the passive limb to the prismatic-universal type limb, the manipulator therefore can have the desired three degrees of freedom, and by applying pure-translational universal joints as three legs instead of the traditional legs, this hybrid robotic mechanism’s stiffness can be greatly improved. Secondly, for the dynamic balancing design approach, the notion of dynamic balancing based on reconfiguration is put forward, which is able avoid the system needing extra counter-devices. In this approach, a counter-mass is not employed but, via reconfiguring the robotic system by shifting the linkages, the robotic system will therefore not get too heavy. On the basis of this concept, firstly we dynamically balance a single limb based on the reconfiguration technique (decomposition) and then integrate the balanced limbs to construct the entire parallel manipulator (integration); i.e., the decomposition and integration concept. Subsequently, for the adaptive control design approach, with the mechanical reconfiguration, the control laws governing the operation of the mechanism also need to be changed. One way to address control system reconfiguration is by applying a “divide and conquer” methodology. This entails modularizing the functionalities: breaking up the control functions into small functional modules, and from those modules assembling the control system according to the changing needs of the mechanism. A hybrid controller for multi-DOF serial robotic manipulators is synthesized through integrating a PID and a model reference adaptive controller. The convergence performance of the PID, MRAC, and PID + MRAC hybrid controllers for a multi-DOF manipulator are compared. The outcome indicates that the convergence speed for the MRAC and PID-MRAC controllers are faster than that of the PID control system, while for the MRAC and PID-MRAC controllers, the convergence performance for the hybrid control system beats the MRAC controller. The combination of a mechatronic design approach and a learning control design approach for robotic mechanisms has great potential for future enhancements. Furthermore, new types of robotic mechanisms synthesis and design for further promoting performance-driven engineering platform remain an open issue.

## Figures and Tables

**Table 1:** Possible solutions of spatial parallel mechanisms.

*F* (DOF of Parallel Manipulator)	$\sum_{k}^{m}C_{k}$ (Total Joint DOF)	For Each *C_k_* (Connectivity Listing)
2	7 × 2 − 6 = 8	($2\leq C_{k}\leq 6$)
		2, 6
		3, 5
		4, 4
3	7 × 3 − 6 = 15	($3\leq C_{k}\leq 6$)
		3, 6, 6
		4, 5, 6
		4, 5, 5
4	7 × 4 − 6 = 22	($4\leq C_{k}\leq 6$)
		4, 6, 6, 6
		5, 5, 6, 6
5	7 × 5 − 6 = 29	($5\leq C_{k}\leq 6$)
		5, 6, 6, 6, 6
6	7 × 6 − 6 = 36	($6\leq C_{k}\leq 6$)
		6, 6, 6, 6, 6, 6

**Table 2:** Feasible limb configurations.

Type	Limb Configuration
120	PUU, UPU, UUP,
	RUU, URU, UUR
201	PPS, PSP, SPP,
	RRS, RSR, SRR,
	PRS, PSR, SPR, RPS, RSP, SRP
310	PPPU, PPUP, PUPP, UPPP,
	RRRU, RRUR, RURR, URRR,
	PRRU, PRUR, PURR, UPRR, RPRU, RPUR, RUPR
500	PPPPP, RRRRR, PRRRR
